# Survey of antimicrobial and probiotic use practices in wildlife rehabilitation in the United States

**DOI:** 10.1371/journal.pone.0308261

**Published:** 2024-08-01

**Authors:** Elizabeth A. Miller, Rachel Amato, Julia B. Ponder, Irene Bueno

**Affiliations:** 1 Department of Veterinary and Biomedical Sciences, College of Veterinary Medicine, University of Minnesota, Saint Paul, Minnesota, United States of America; 2 The Raptor Center, College of Veterinary Medicine, University of Minnesota, Saint Paul, Minnesota, United States of America; 3 Department of Veterinary Population Medicine, College of Veterinary Medicine, University of Minnesota, Saint Paul, Minnesota, United States of America; 4 Bristol Veterinary School, University of Bristol, Langford, Bristol, United Kingdom; Universidad Autonoma de Chihuahua, MEXICO

## Abstract

Antimicrobial resistance is a global health concern. As such, there have been increased efforts to monitor and standardize antimicrobial prescribing practices in humans and domestic animals. In contrast, there is relatively little known about specific prescribing practices in wild animals despite the wide use of antimicrobials and other microbial interventions, such as probiotics to treat captive wildlife. Therefore, the goal of this study was to examine current antimicrobial and probiotic use from a cross-section of wildlife rehabilitation facilities in the United States. An anonymous electronic survey was sent to 105 United States permitted wildlife facilities to collect information about admissions, current antimicrobial and probiotic use practices, and current staff knowledge and attitudes surrounding antimicrobial resistance and probiotic effectiveness. Respondents from over 50% of facilities participated in the survey (54/105), including 45 facilities that treated birds. All facilities reported using antimicrobials, including some from groups considered critically important for human medicine, for a wide range of medical conditions and prophylaxis. Among antibiotics, enrofloxacin and amoxicillin-clavulanic acid were the most commonly used. Antifungals were not as widespread, but itraconazole was the most commonly used. Over 75% of respondents said that their facilities would benefit from having standardized antimicrobial guidelines in place. Probiotics were also used in more than 50% of facilities, but there was notable disparity in opinions regarding their efficacy. The results of this survey are a first step towards understanding antimicrobial and probiotic use practices in the treatment of captive wildlife and developing an antimicrobial stewardship program for wildlife rehabilitation.

## Introduction

Antimicrobial resistance (AMR) is a global public health problem. One of the main drivers of AMR is the “misuse” of antimicrobials in humans and animals, as antimicrobials can exert selection pressures which may promote the emergence and spread of antimicrobial resistant bacteria [1]. This relationship underscores the need for antimicrobial stewardship–a coordinated effort to promote the judicial use of antimicrobial agents in human and animal healthcare settings via policies, guidelines, and interventions [2, 3]. Specifically, this involves strategies aimed at optimizing antimicrobial therapy to achieve the best clinical outcomes while minimizing the emergence of AMR and reducing the risks of adverse effects. By emphasizing the importance of evidence-based prescribing practices, surveillance, and education, antimicrobial stewardship programs play a crucial role in combating AMR and preserving the effectiveness of antimicrobial agents for future generations [2, 3].In accordance with the objectives of antimicrobial stewardship, there has been a recent increase in published surveys of human and animal medical practitioners assessing antimicrobial use (AMU) and awareness and understanding of AMR (e.g., [4–6]). For animal-based surveys, the main focus has either been on livestock due to the direct relationship with humans through the food chain (e.g., [7, 8]), or companion animals (namely dogs and cats) (e.g., [9–11]). In contrast, there has been little effort to gather information on the practices and opinions of individuals providing veterinary care to wildlife. Wildlife undergo medical care in permanent settings such as zoo collections or sanctuaries, or in more temporary situations such as wildlife rehabilitation facilities where the ultimate goal is the release of these animals back to the wild [12]. In the United States (U.S.) alone, it is estimated that hundreds of thousands of sick, injured, or orphaned animals are presented for rehabilitation every year [12]. The size of the operations, level and experience of professionals involved, and the regulatory environment vary widely. Specifically, facilities can range from small, local operations run by volunteers or a few staff members, to large facilities with extensive resources and professional staff. Funding sources also vary, with most facilities relying on small public donations or grants, while others may also receive government support or operate as part of larger, often non-profit, organizations. These differences may significantly impact AMU practices, as larger, better-funded facilities may have more resources to implement comprehensive antimicrobial stewardship programs, while smaller facilities with limited funding may face greater challenges in accessing and implementing them. Guidelines put forward by the International Wildlife Rehabilitation Council and National Wildlife Rehabilitators Association acknowledge that in most states, veterinarians are legally responsible for prescribing medical care for wildlife patients and can delegate some responsibilities to rehabilitators through “mutually agreeable” written protocols [13]. However, these guidelines do not include specific treatment protocols, such as which antibiotics to use at what dosage, frequency, and duration for which circumstances. Thus, AMU may vary significantly between rehabilitation facilities based on individual preferences and experiences of the attending veterinarian(s) and staff. The potential diversity in AMU practices within wildlife rehabilitation facilities prompts critical examination of the possible implications, including the selection for AMR and subsequent introduction of antimicrobial resistant bacteria (ARB) into natural ecosystems upon release of the rehabilitated host [14]. Indeed, wild animals may act as reservoirs for AMR and are thought to contribute to the spread of AMR in the environment [14–16]. Birds, in particular, are hypothesized to play a role in AMR dissemination as many species are in close contact with anthropogenic environments (e.g., gulls, Canada geese) and can travel vast geographic distances during migration [14–16]. Currently, AMR has been studied in wildlife rehabilitation settings in different parts of the world, highlighting the role these facilities play in terms of AMR surveillance (e.g., [17–20]). However, our understanding of the possible contribution of rehabilitation facilities to AMR emergence and spread into the wide environment is lacking. A further consideration of AMU in rehabilitation settings is the negative impact antimicrobials may have on the host gut microbial community (i.e., the “gut microbiome”) [21]. Antimicrobial therapy can lead to a reduction in microbial diversity, altered composition, and selection for resistant opportunistic pathogens, which may alter host digestion, nutrient absorption, and immunity homeostasis [22, 23]. Ultimately, these changes to the gut microbiome could affect the health and fitness of the reintroduced wild animal [21]. In an effort to mitigate the negative side effects of antimicrobials and improve overall host health, some rehabilitation facilities employ probiotics, which are defined as “live microorganisms that confer health benefits to the host when administered in adequate amounts” [24]. Probiotics have been shown to enhance overall well-being and resilience in both domestic and wild animals [25, 26] and have demonstrated great potential in disease mitigation and reducing the negative effects of stress, environmental changes, and antimicrobial treatments on wild animals [25, 27, 28]. However, similar to the lack of standardized AMU guidance, there are no established protocols or guidelines regarding the use of probiotics in wildlife rehabilitation facilities. Given the limited availability of data concerning the application of antimicrobials and probiotics in wildlife rehabilitation and the importance of antimicrobial stewardship for ongoing efforts to combat AMR, the goal of this study was to investigate antimicrobial and probiotic use practices within wildlife rehabilitation facilities in the United States. Due to the potential role birds play in the dissemination of AMR and the fact that they comprise approximately half of all cases at wildlife rehabilitation facilities [29, 30], particular focus was given to facilities treating birds. The findings of this survey represent a first step towards understanding trends in antimicrobial and probiotic use in wildlife rehabilitation and can serve as a foundation for the development of antimicrobial stewardship initiatives tailored to treatment of captive wildlife.

## Materials and methods

### Ethics approval

This study was approved by the Institutional Review Board (IRB) of the University of Minnesota (STUDY00015087). Answers to the survey questions were anonymous.

### Study design

A cross-sectional survey was conducted in 2022 (February-March) to obtain data on antimicrobial and probiotic use within U.S. wildlife rehabilitation. The inclusion criteria for survey recipients consisted of a) permitted U.S. wildlife rehabilitators, b) wildlife facilities that admitted birds, and c) wildlife facilities that were not volunteer run. The list of recipients was obtained through the ‘Partners for Wildlife’ program [31] and reflected all the wildlife rehabilitation facilities in the program’s seven state focus area (WI, MN, ND, ID, MT, WA, AK) that met the inclusion criteria. The survey was intended to capture information per wildlife facility, so if there were several permit holders within one facility, only one of them was requested to answer the survey. The survey was written in English by the research team (EAM, RA, JP, IB), designed using Web-based survey software (Qualtrics, Provo, UT), and consisted of five sections (general information, antibiotic and antifungal use, disinfectant use, probiotic use, and perceptions about AMR and antimicrobial use) for a total of 26 questions. The questions included open-ended, multiple choice, yes/no, and 5-point Likert scale (from strongly agree to strongly disagree). The approximate time to complete the survey was estimated to be between 15 and 20 minutes. None of the questions were set as mandatory to be completed and questions could be skipped. An email containing a cover letter explaining the project and a link to the survey in Qualtrics was sent to the survey recipients. An option to send the survey in paper format was also provided. The complete survey is available as supplemental material to this manuscript (S1 File). Prior to sending the final version, the survey tool was beta tested by five wildlife rehabilitators and subsequently improved based on their feedback. After sending the final survey (launched on February 21st, 2022), two weekly email reminders were sent to recipients, allowing a total of three weeks to submit responses.

### Data analyses

All data analyses were conducted within R, version 4.1.2 (R Core Team, 2020). Due to the small number of responses in some categories, response variables for some questions were recoded as either fewer categorical variables or a binary ‘Yes/No’ response. Average annual bird admission number was used as a proxy for overall facility size, with admission ranges grouped into three categories (i.e., sizes) for further analyses: small (0–50 birds admitted annually), medium (51–500 birds admitted annually), and large (501–5000 birds admitted annually). These groupings were based on how the Partners for Wildlife initiative categorizes facilities in their database and generally reflects size of facilities (individual home rehabilitator, small volunteer-led facilities and larger centers with paid staff).

The linear-by-linear association test with an asymptotic approximation of the exact distribution was used to test for a linear trend between two ordered categorical variables or between ordered categorical and binary variables. Tests were conducted using the *lbl_test*() function from the R package coin, version 1.4–2 [32]. Pearson’s chi-square test with an asymptotic approximation of the exact distribution was used to test for a trend between two binary variables. Tests were conducted using the *chisq_test*() function from the R package coin. Statistical significance was defined with an ⍺ level of 5% and Benjamini-Hochberg adjusted P-values were calculated to correct for multiple testing where applicable. All plots were generated using the R package ggplot2, version 3.3.6 [33].

## Results

Invitations to participate in the study were sent to 105 permitted wildlife rehabilitation facilities. A total of 54 responses were received, for a response rate of 51.4% (54/105). The survey was anonymous, and thus no identifiable information regarding individual characteristics of the respondents (e.g., demographics) or the wildlife facility itself (e.g., location) were included in the questionnaire.

Of the 54 responses, 46 (85.2%) treated birds, 33 (61.1%) treated mammals, 21 (38.9%) treated reptiles, 16 (29.6%) treated amphibians, and two (3.7%) treated invertebrates. None of the facilities treated fish. One facility did not respond to all the survey questions and was thus excluded from further analyses. Given the proposed importance of wild birds in the spread of AMR, we pared our dataset down to the 45 facilities that treated birds either exclusively or in addition to other animals (henceforth referred to as “all-taxa facilities”). Of those 45 facilities, 18 (40.0%) only treated birds (henceforth referred to as “bird-only facilities”), while the remaining 27 also treated mammals (n = 26; 57.8%), amphibians (n = 15; 33.3%), reptiles (n = 19; 42.2%), and/or invertebrates (n = 2; 4.44%) (Table 1). Facilities treated a range of bird groups, with 41 (91.1%) treating raptors, 38 (84.4%) small birds, 29 (64.4%) waterfowl, 27 (60%) shorebirds, and one (2.2%) seabirds. There were nine facilities that limited specialized treatment to treating only one group of birds: raptors (n = 7; 15.56%) and small birds (n = 2; 4.44%).

**Table 1:** General information about the wildlife rehabilitation facilities included in the survey.

Characteristic	All-taxa facilitiesª		Bird-only facilities	
	(n = 45)		(n = 18)	
	n	%	n	%
Type of wildlife				
Birds	45	100	-	-
Mammals	26	57.8	-	-
Amphibians	15	33.3	-	-
Reptiles	19	42.2	-	-
Invertebrates	2	4.44	-	-
Fish	0	0.00	-	-
Annual bird admissions range ^b^				
0–10	5	11.1	2	11.1
11–50	3	6.67	2	11.1
51–100	8	17.8	3	16.7
101–500	15	33.3	6	33.3
501–1000	6	13.3	3	16.7
1001–5000	8	17.8	2	11.1
Access to veterinarian(s)				
Yes	43	95.6	17	94.4
No	2	4.44	1	5.56

ªFacilities that treated birds *in addition to* other animals, including mammals, amphibians, reptiles, and/or invertebrates.

^b^Bird admission number ranges were grouped into three categories for statistical analyses: small (0–10, 11–50), medium (51–100, 101–500), and large (501–1000, 1001–5000).

When asked about the average number of bird admissions in a typical pre-pandemic year, the ranges varied from 1–10 birds annually (n = 5; 11.1%) up to 1001–5000 birds annually (n = 8; 17.8%) (Table 1). The majority of facilities received between 101–500 birds annually (n = 15; 33.3%). Access to one or more veterinarians was reported by all but two (n = 43; 95.6%) of the facilities (Table 1), with both facilities without veterinary access reporting annual bird admissions between 51–100 birds.

### Antimicrobial use

All 45 facilities (100%) reported use of antimicrobials (antibiotics, antifungals, and/or antivirals) for treatment of admitted animals. Of those, 62.2% (28/45) had antibiotic use protocols written by veterinarians (Table 2). For the subset of bird-only facilities, antibiotic use protocols were reported by 50.0% (9/18) of respondents. Overall, large facilities (based on reported annual bird admission numbers) were significantly more likely to have antibiotic use protocols than medium or small-sized facilities (all-taxa: *p* = 0.020, bird-only: *p* = 0.10).

**Table 2:** Antimicrobial use in wildlife rehabilitation facilities included in the survey.

Information	All-taxa facilitiesª		Bird-only facilities	
	(n = 45)		(n = 18)	
	n	%	n	%
Written antibiotic use protocols				
Yes	28	62.2	9	50.0
No	17	37.8	9	50.0
Written antifungal use protocols				
Yes	20	44.4	7	38.9
No	25	55.6	11	61.1
AMU expenses				
0–5%	24	53.3	10	55.6
6–10%	5	11.1	2	11.1
11–30%	5	11.1	2	11.1
Unknown	11	24.4	4	22.2
Bacterial culture and sensitivity testing				
Yes	30	66.7	9	50.0
No	15	33.3	9	50.0
Factors affecting decision-making to stop AMU				
Adverse reaction	27	60.0	10	55.6
Antimicrobial resistance	18	40.0	7	38.9
Bacterial culture/sensitivity results	19	42.2	4	22.2
Cost	6	13.3	1	5.60
Veterinarian recommendation	1	2.22	0	0.00
Pre-made protocol by veterinarian	19	42.2	5	27.8
Recommended duration	40	88.9	16	88.9
Resolution of clinical signs	34	75.6	13	72.2
Source of information about AMU				
Continuing education courses	36	80.0	14	77.8
Internet	30	66.7	10	55.6
Other rehabilitators	29	64.4	9	50.0
Peer reviewed literature	31	68.9	10	55.6
Social media	0	0.00	0	0.00
Veterinarians	45	100	18	100

AMU, antimicrobial use.

ªFacilities that treated birds *in addition to* other animals, including mammals, reptiles, amphibians, and/or invertebrates

Compared to antibiotic protocols, written protocols for antifungal use were less common, with 44.4% (20/45) of all-taxa facilities and 38.9% (7/18) of bird-only facilities having them in place (Table 2). Bird admissions were significantly associated with the presence of antifungal use protocols, with larger facilities more likely to have antifungal use protocols than smaller facilities (all-taxa: *p* = 0.0015, bird-only: *p* = 0.082).

All facilities (100%) reported obtaining information about antibiotic and antifungal use from veterinarians, but other sources for information were also used, including peer-reviewed literature (all-taxa: 68.9%; bird-only: 55.6%), continuing education literature (all-taxa: 80.0%; bird-only: 77.8%), other rehabilitators (all-taxa: 64.4%; bird-only: 50.0%), and the internet (all-taxa: 66.7%; bird-only: 55.6%) (Table 2).

**Fig 1:**
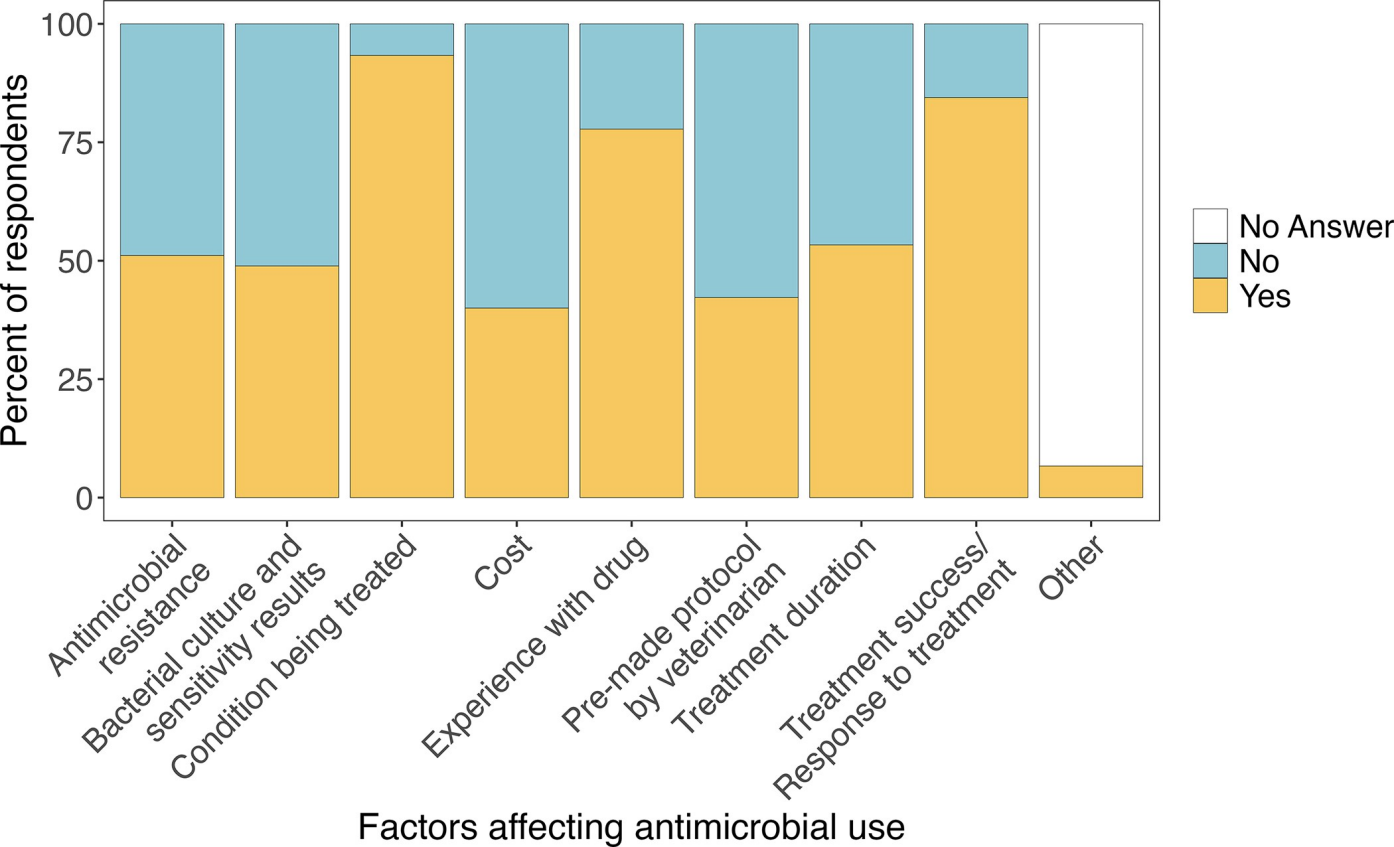
Factors that contributed to wildlife rehabilitation facilities decision to use antimicrobials.

Respondents reported a number of factors that contributed to their facility’s decision-making concerning use of antimicrobials, with condition treated cited as the most common factor across both all-taxa (93.3%; 42/45) and birds-only (88.9%; 16/18) facilities (Fig 1).While 66.7% (30/45) of the all-taxa facilities (50.0% [9/18] of bird-only facilities) reported performing some capacity of bacterial culture and sensitivity diagnostic testing, not all of those facilities said bacterial culture and sensitivity results influenced their decision to use antimicrobials (all-taxa: 48.9% [22/45]; bird-only: 27.8% [5/18]). Cost was also reported as a factor to be considered for 40.0% (18/45) of all-taxa facilities and 38.9% (7/18) of bird-only facilities.

**Fig 2:**
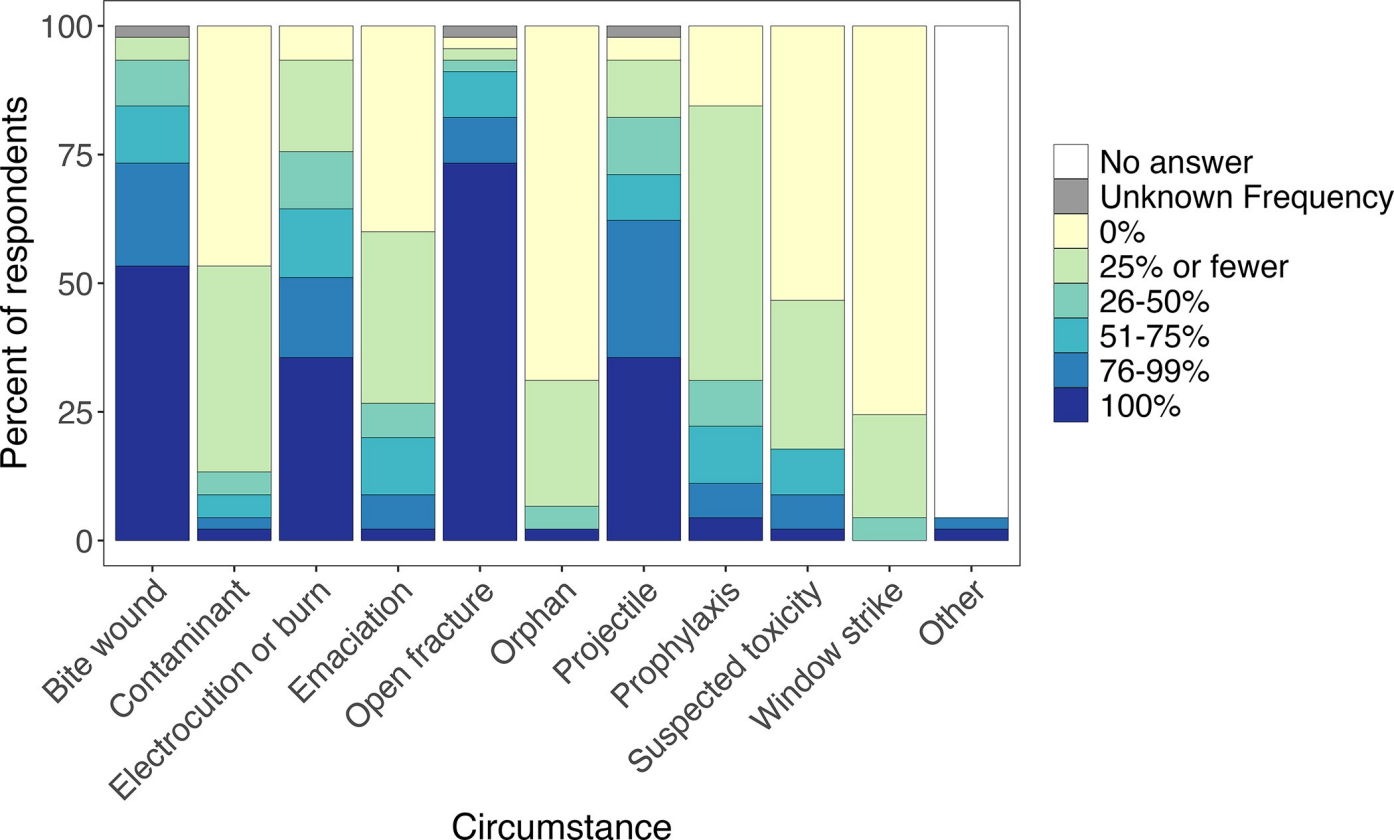
Conditions for which wildlife rehabilitation facilities used antibiotics.

The conditions most commonly treated with antibiotics were bite wounds and open fractures, with 53.3% (24/45) and 73.3% (33/45) of facilities, respectively, using antibiotics in 100% of these circumstances (44.4% and 72.22% for bird-only facilities) (Fig 2). More than 20% (10/45) of all facilities (33.3% [6/18] of bird-only facilities) reported using antibiotics for prophylaxis in at least 50% of cases. Only 15.6% (7/45) of facilities (27.8% [5/18] of bird-only facilities) reported never using antibiotics for prophylaxis. Size of the facility was not significantly associated with the frequency antibiotics were used as treatment for any of the listed conditions (all adjusted *p*>0.05).

Overall use of antifungals was lower than use of antibiotics. The condition most commonly treated with antifungals was respiratory diseases, with 11.1% (5/45) of all-taxa facilities (11.1% [2/18] of bird-only facilities) using them in 100% of these cases. Use of antifungals for prophylaxis was considerably less common than for antibiotics with only 40.0% (18/45) of facilities (50.0% [9/18] of bird-only facilities) reporting never using antifungals for prophylaxis. Further, larger facilities were significantly more likely to use antifungals for prophylaxis (adjusted *p* = 0.021) and in cases of emaciation (adjusted *p* = 0.023).The factors most commonly reported as affecting a facility’s decision to stop antimicrobial treatment were the recommended duration (all-taxa: 88.9% [40/45]; bird-only: 88.9% [16/18]) followed by the resolution of clinical signs (all-taxa: 75.6% [34/45]; bird-only: 72.2% [13/18]) (Table 2). Cost was indicated as a factor affecting when to stop antimicrobial treatment for 13.3% (6/45) of all facilities (5.6% [1/18] of all-bird facilities). Larger facilities were significantly more likely to consider veterinarian-written AMU protocols with set time frames than smaller facilities (adjusted *p* = 0.034).

**Fig 3:**
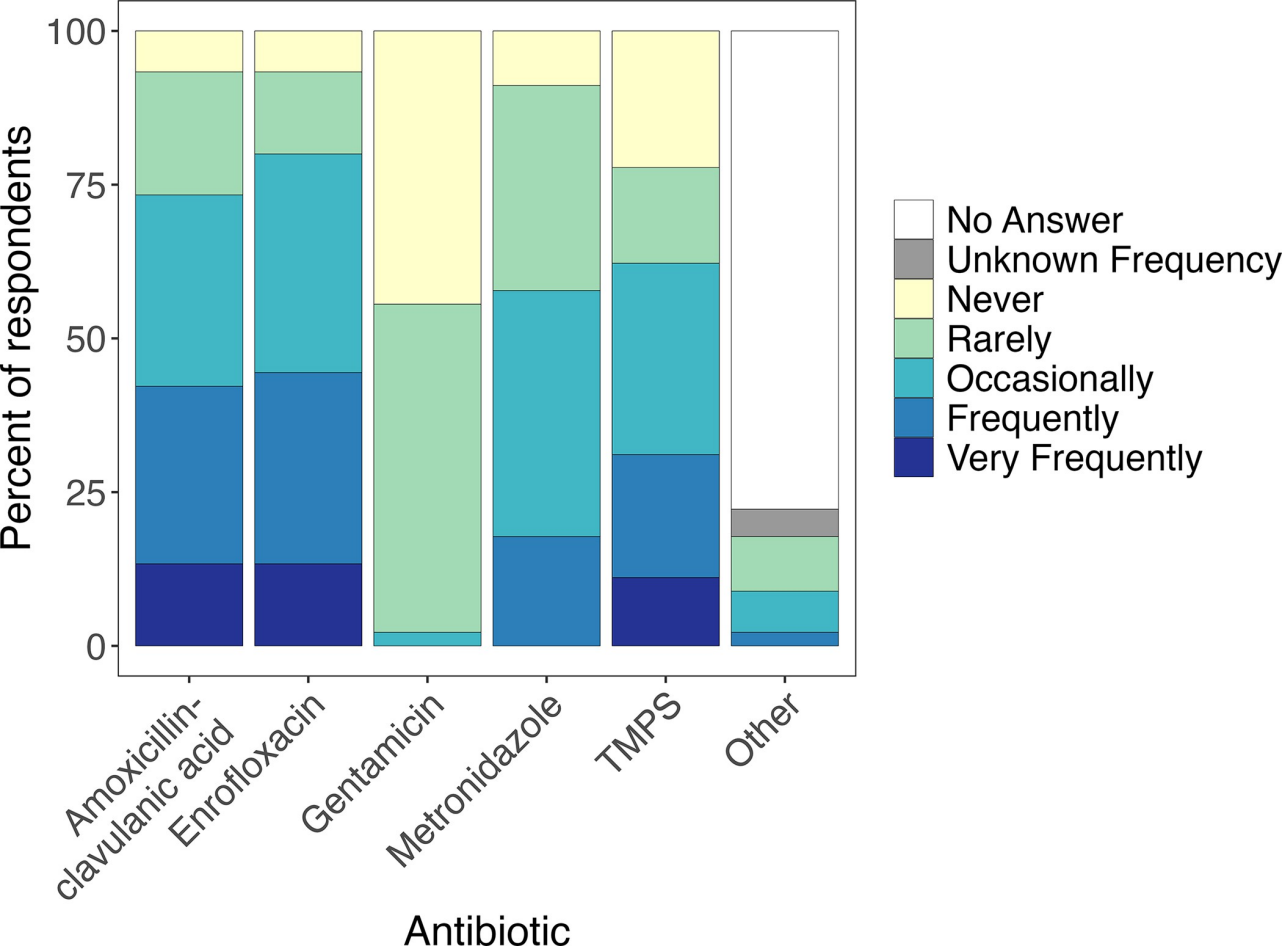
Antibiotics used by the wildlife rehabilitation facilities.

The most commonly used antibiotic drugs were enrofloxacin and amoxicillin-clavulanic acid, with more than 40% of all-taxa facilities (over 30% of bird-only facilities) reporting use of them “frequently” or “very frequently” (Fig 3). Trimethoprim/sulfamethoxazole (TMPS) was also used “frequently” or “very frequently” in over 30% of all-taxa facilities (16.7% of all-bird facilities). Gentamicin was either “rarely” used (all-taxa: 53.3% [24/45]; bird-only: 38.9% [7/18]) or “never” used (all-taxa: 44.4% [20/45]; bird-only: 61.1% [11/18]). Larger facilities were significantly more likely than medium or small ones to use amoxicillin-clavulanic acid, TMPS, and metronidazole (all adjusted *p* = 0.041).Additional antibiotics listed by respondents in the “Other” category included third-generation cephalosporins (n = 4), penicillin (n = 2), and clindamycin (n = 5).

For antifungals, itraconazole was the most commonly used, with 4.4% (2/45) of facilities using it “very frequently”, 15.6% (7/45) using “frequently”, and 37.8% (17/45) using “occasionally” (5.6%, 11.1%, and 27.8% for bird-only facilities, respectively). Other listed antifungals, including voriconazole, terbinafine, and nystatin, were typically used (>50% of facilities) “rarely” or “never” by facilities.

### Probiotic use

**Fig 4:**
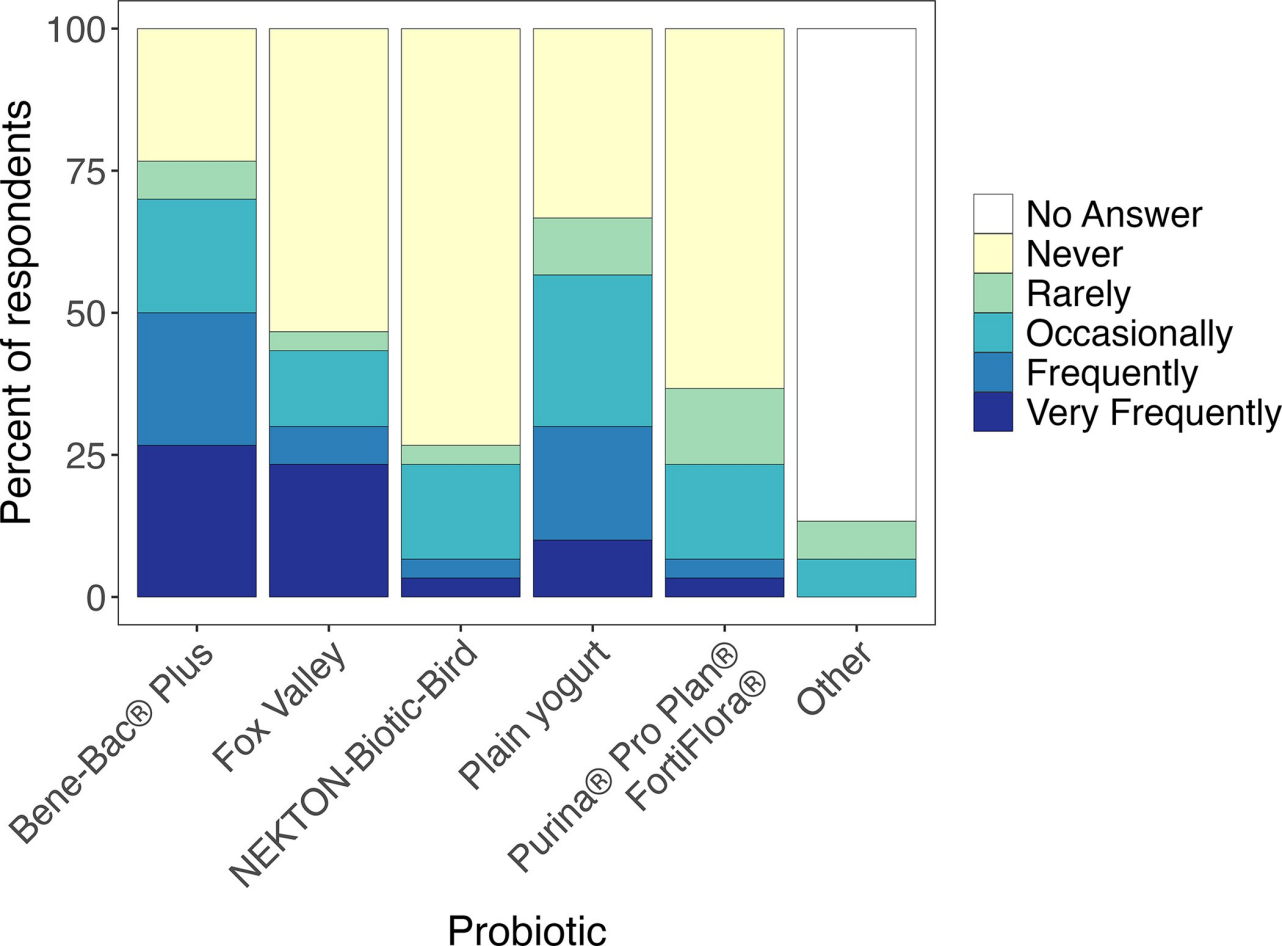
Probiotics used by the wildlife rehabilitation facilities.

**Fig 5:**
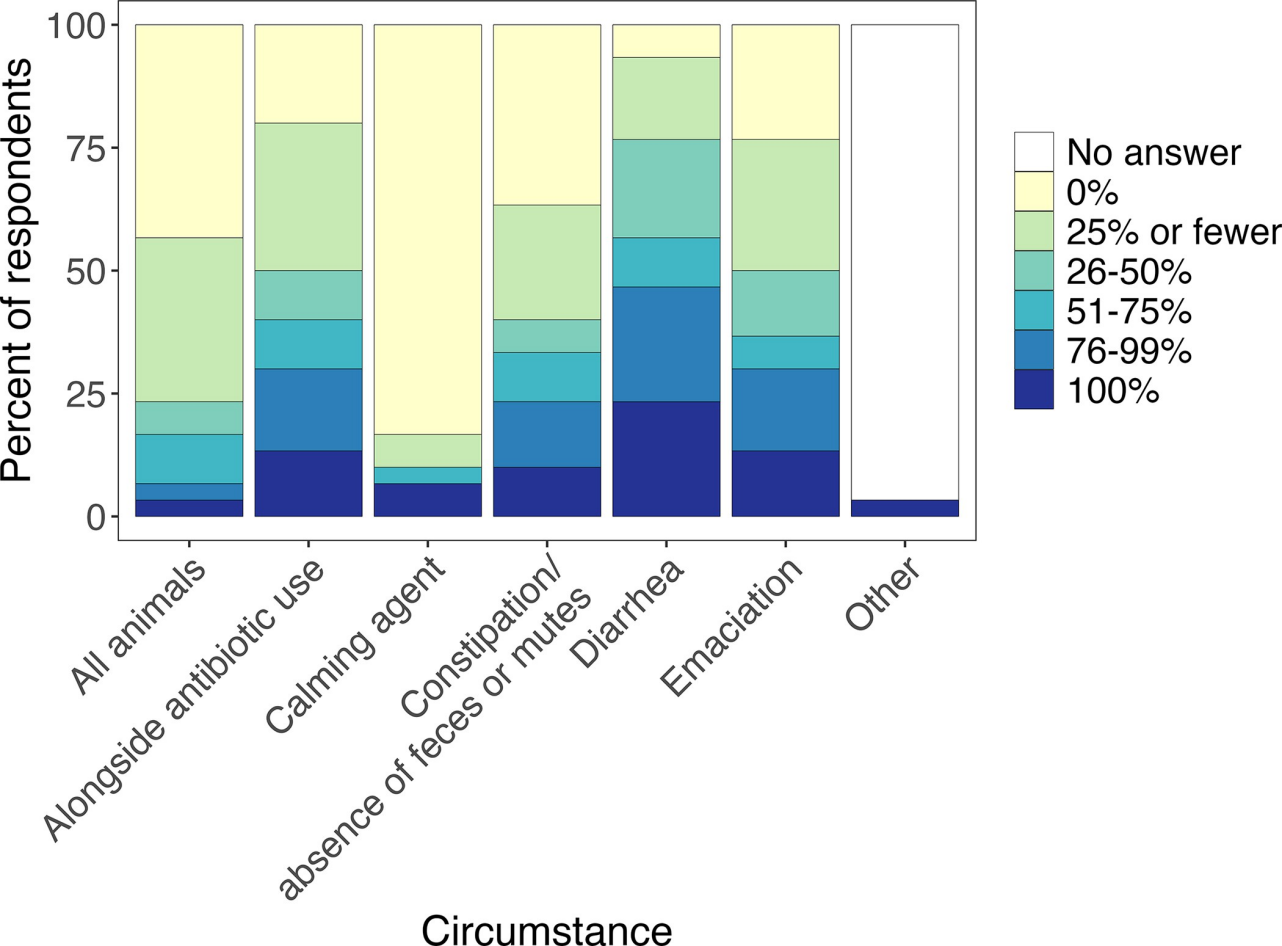
Conditions for which wildlife rehabilitation facilities used probiotics.

Out of the 45 facilities, 66.7% (30/45) reported using probiotics, including 50% (9/18) of the bird-only facilities. Of those facilities that used probiotics, the most commonly used (“frequently” or “very frequently”) were PetAg Bene-Bac Plus® (all-taxa: 50%; bird-only: 33.3%), followed by Fox Valley (all-taxa: 30%; bird-only: 0%) (Fig 4). Plain yogurt was also used “frequently” or “very frequently” by 30% of facilities (11.1% of all-bird facilities). The most frequent use of probiotics was for diarrhea and emaciation, followed by concurrent antibiotic use and constipation (Fig 5). Five facilities (16.7%), including two all-bird facilities (22.2%), reported giving probiotics in some capacity as a calming agent.

### Disinfectant use

All 45 facilities reported using disinfectants for cleaning. Among the disinfectant types, 11 facilities reported using at least one type of quaternary ammonium compound (QAC), specifically Roccal D (n = 5, 11.6%), Parvosol (n = 3, 6.9%), Kennel Kare (n = 1, 2.3%), Chlorhexidine (n = 2, 4.7%), HDQ Neutral (n = 1, 2.3%), Basic-G (n = 1, 2.3%), and KennelSol (n = 1, 2.3%). The majority of those facilities used the QACs daily (n = 8, 72.7%).

### Perception and knowledge about AMR and AMU

**Fig 6:**
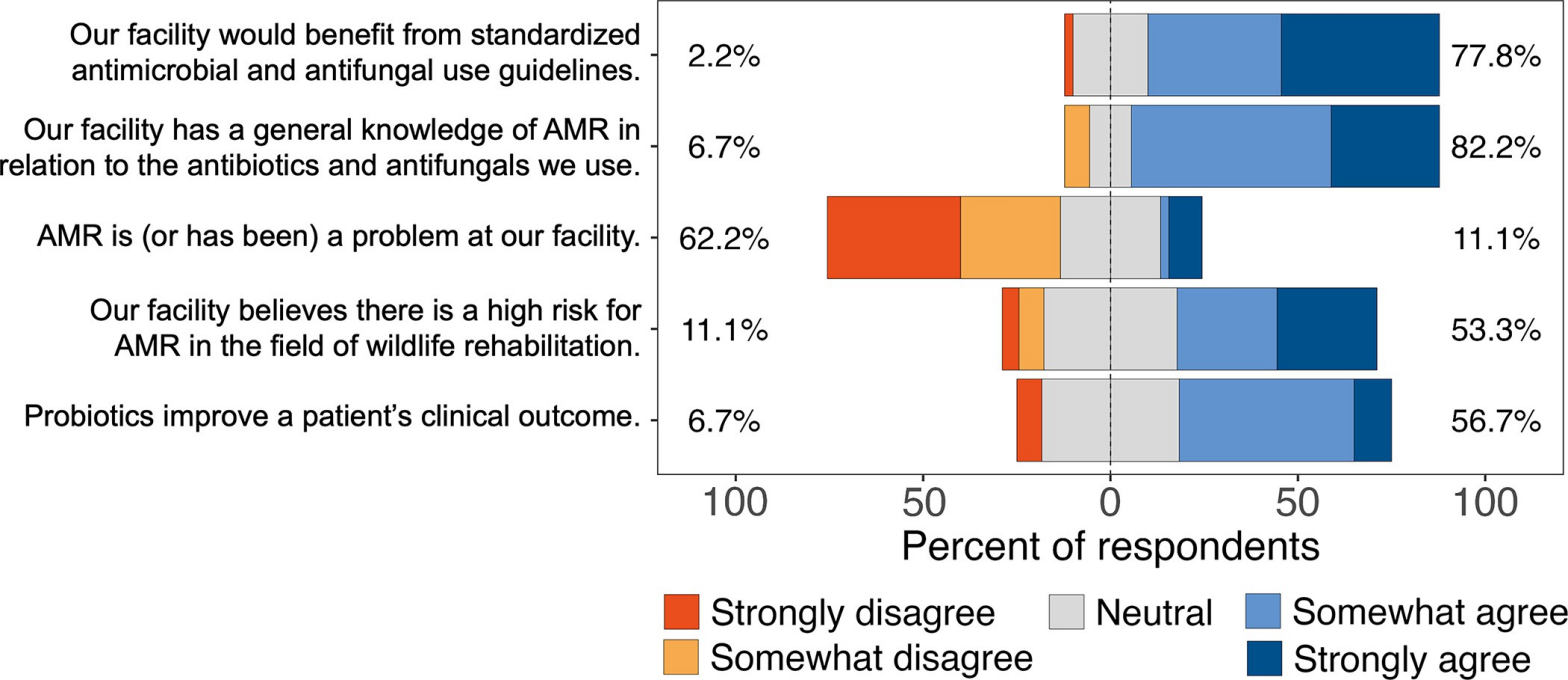
Respondents’ opinions about different aspects related to antimicrobial resistance and antimicrobial and probiotic use in wildlife rehabilitation.

Out of the 45 respondents, 77.8% (35/45) “somewhat agreed” or “strongly agreed” that their facility would benefit from having standardized antimicrobial and antifungal guidelines (Fig 6). When asked if the facility had general knowledge of AMR in relation to antibiotic and antifungal use, 82.2% (37/45) “somewhat agreed” or “strongly agreed” with the statement. Interestingly, while only 11.1% (5/45) agreed (“strongly” or “somewhat”) that AMR was a problem at their facility, 53.3% (24/45) agreed that AMR posed a high risk for the wildlife rehabilitation field as a whole. Of the 30 respondents who reported using probiotics in their facilities, 56.7% (17/30) agreed that probiotics improve a patient’s clinical outcome.

## Discussion

In this study, we used a cross-sectional survey to investigate antimicrobial and probiotic use practices in wildlife rehabilitation in the U.S., with special focus on those facilities that treated birds. The response rate of 51.4% is fairly decent and can even be considered above average for online surveys [34]. However, this response rate could have been influenced by the enhanced commitment of wildlife rehabilitators toward improving animal welfare, as well as AMR being a frequently discussed topic within the sector. Indeed, given participation in the survey was voluntary, one of the main limitations of this study is the potential overrepresentation of facilities with stronger interest in the topics of AMU, AMR, and probiotics. Results may also not be representative of the entire U.S. rehabilitation community given that wildlife facilities were selected based on specific criteria (i.e., permitted, not volunteer run), which may have restricted inclusion to those with more veterinary access. The focus on rehabilitation facilities that admit birds could also have skewed our findings, potentially overlooking valuable data from other facilities, such as those that only treat mammals. That being said, the subset of 45 wildlife rehabilitation facilities we included in our study reported a wide range of practices and perspectives, offering valuable insights despite potential limitations in generalizability to the entire U.S. (and global) rehabilitation community. In our study, antimicrobials (specifically antibiotics and antifungals) were used in all facilities with more or less frequency and for a variety of reasons. The reported use of antimicrobials prophylactically was of particular interest given the general veterinary recommendation to avoid prophylactic use of antimicrobials whenever possible [35]. Among specific antibiotics, amoxicillin-clavulanic acid and enrofloxacin were routinely used across all facilities. These are among the most commonly used antibiotics in small animals worldwide [36] and have been reported as frequently used by veterinarians in other surveys as well [10]. Enrofloxacin in particular, is widely prescribed for the treatment of both domestic and exotic pet birds [37–39]. This drug belongs to the fluoroquinolone class, and although it is not used in humans, similar compounds within the same class (e.g., ciprofloxacin) are classified under the ‘Watch’ category established in the AWaRe (Access, Watch, Reserve) classification by WHO [40]. This group includes antibiotics considered ‘Critically Important for Human Medicine’ and/or those that have a higher potential of selection of ARB [40]. Within the U.S., the use of fluoroquinolones is highly restricted in animals and is not approved for any extra label use in food-producing animals due to concerns of increasing antimicrobial resistant pathogens [41]. This includes prohibition of fluoroquinolones such as enrofloxacin in rehabilitated wild animals if the animal “has any possibility of being hunted or harvested for food by a human” (i.e., minor food animal species) [42, 43].

Under the same regulations, use of metronidazole in wild minor food animal species is also prohibited [42, 43], yet more than 90% of surveyed facilities reported using the drug. Three facilities also reported using third-generation cephalosporins, which are permitted for use in wild animals in the U.S. [42, 43], but do fall within the WHO’s AWaRe ‘Watch’ category [40]. The remaining antibiotics fell into the AWaRe ‘Access’ category, which are those drugs that show lower antibiotic resistance potential [40]. While some U.S. rehabilitation facilities have stopped all use of restricted drugs in response to government regulations, the high number of facilities reporting the use of enrofloxacin and metronidazole in the current study suggests there is clearly a need to reevaluate some antibiotic usage practices within rehabilitation facilities. A limitation of this study is that we did not ask respondents to identify which species they use various antimicrobials in or their familiarity with restrictions in wild minor food animal species.

Interestingly, approximately one third of facilities reported not performing any bacterial culture and sensitivity testing. An additional 51% of facilities that did conduct bacterial culture and sensitivity testing said that the results of those diagnostics were not considered when making decisions concerning antimicrobial use. Antimicrobial sensitivity testing determines the effectiveness of the chosen antimicrobials against the bacterial strains cultured. This information is not only vital for accurate diagnosis and provision of appropriate targeted treatments for patients but is also necessary information for developing appropriate antimicrobial use guidelines [44].

In addition to antibiotic use, results from this survey also provided a general overview of antifungal and QAC applications in rehabilitation facilities. Among antifungals, itraconazole use was frequently reported. This drug is routinely used for prevention and treatment of aspergillosis, one of the most common fungal diseases affecting avian species in captivity [45–47]. This likely explains why our survey found that facilities with higher bird admissions were more likely to use antifungals for prophylaxis and emaciation and were more likely to have antifungal protocols in place compared to facilities with fewer bird admissions. Even though research and AMR guidelines have mostly focused on resistant bacteria, antifungal resistance is a rising concern in human health [48]. Yet, there is limited understanding of the potential emergence of antifungal resistance, particularly concerning the role that wild birds might play in disseminating azole-resistant strains (azoles such as itraconazole) [49].

For QACs, only about 25% of respondents reported using QAC disinfectants to clean their facilities. QACs are common ingredients in cleaning products, such as disinfectants, due to their broad-spectrum antimicrobial properties [50]. However, QACs can potentially select and enrich for ARB through different mechanisms, sparking concern over possible adverse environmental and human health impacts [50–52]. While real-world evidence for the use of QAC disinfectants promoting AMR emergence is still quite sparse [51], QACs should be considered in future assessments of AMR in wildlife rehabilitation until more comprehensive studies can provide a better understanding of the link between QACs and AMR.

To date, there are no antimicrobial stewardship policies specific to wildlife species in rehabilitation settings. Yet AMU guidelines would be beneficial given that animals often present with severe traumatic injuries, requiring intensive treatment frequently requiring antimicrobials. This was highlighted in the survey by the high frequency some case types were treated with antibiotics, including bite wounds, open fractures, projectiles, and burns. Examples of AMU guidelines are becoming increasingly common in veterinary settings, especially for treatment of food-producing and small domestic animals [35, 53]. Further, the evaluation of antimicrobial prescribing practices before and after implementation of such guidelines shows a decline in AMU and a shift towards the use of less clinically important antibiotics [54–56]. The wide range of AMU practices observed in this study suggests there is a need to develop standardized AMU guidelines specifically for wildlife rehabilitation settings. This is highlighted by our findings that larger facilities, which may have access to more resources, were more likely to have and utilize AMU protocols written by veterinarians than smaller facilities. Overall, given the high number of respondents who said their facilities would benefit from standardized antimicrobial and antifungal guidelines, we believe formal AMU guidelines would be well-received within the entire rehabilitation community.

Probiotic use was reported in more than half of the facilities surveyed. Probiotic therapy is increasingly used in human medicine and domestic animals to modulate the gut microbiota and host immune system with the goal of improving host health [57–59]. In particular, probiotics can be used to treat specific diseases as an alternative to antibiotics or chemotherapy or used in conjunction with antibiotics to mitigate the negative gastrointestinal side effects [60, 61]. In food animals, including ruminants, poultry, and swine, probiotics are frequently used as nutritional supplements or feed additives for both disease prevention and improved performance, such as growth promotion [57, 61, 62]. Similarly, there are a wide range of commercial probiotic products available for use in companion animals, particularly dogs and cats, for a variety of health benefits [59]. In contrast, research of probiotic applications in wild animals is in the early stages of validation, standardization in approaches, and risk assessment [27]. A small number of studies provide promising evidence that probiotics can be used for wildlife disease treatment and management. Laboratory assessments and field trials in bees, bats, and amphibians showed probiotic applications can lead to host improvements in immune response, increased survivorship, and reduced pathogen load and disease severity [28, 63]. Further, data extrapolated from humans and domestic animals suggest probiotics may be useful in wild animals for mitigating the negative effects of captivity including environmental and dietary changes, stress, and antimicrobial treatments [27, 28]. Among the facilities surveyed in this study, probiotics were reportedly used for a wide range of circumstances, from treatment of diarrhea to calming agents. Yet only 56.7% (17/30) of those agreed that probiotics had a positive clinical outcome in their patients. The remaining facilities using probiotics either were neutral or strongly disagreed with probiotic effectiveness. This disjuncture between facility probiotic practices and practitioner beliefs warrants further attention in the future.

The specific types of probiotics facilities reported using are also worth addressing. When selecting probiotic candidates, host specificity is an important criterion due to host differences in anatomy (e.g. complexity of GI tract), metabolism, immune function, and resident microbiota [64, 65]. Bacterial strains that have coevolved with a host species may be better able to colonize and persist in that respective host compared to strains derived from distantly related species [66, 67]. Probiotics used by the facilities appeared to be largely non-host-specific and thus it is unclear whether they are suitable for the animal species being treated. For example, 11 facilities, including three bird-only facilities, reported using Purina® Pro Plan® FortiFlora® probiotics, which include products formulated for use in canines and felines. To our knowledge, there are no studies of these products in other species, including small wild mammals and birds. Plain yogurt was also reportedly used in 20 facilities, including four all-bird facilities. However, standard yogurt likely provides little benefit to the host because the starter bacteria (*Lactobacillus bulgaricus* and *Streptococcus thermophilus*) typically do not survive and colonize the GI tract [68]. Further, while there is some evidence that commercially available probiotic-enriched yogurt is beneficial for human health [69], there has been little investigation into the efficacy of yogurt as a probiotic in other species beyond animal models and poultry [70–73]. In the best-case scenario, administration of unsuitable probiotics may simply provide no benefit to the treated host. In the worst case, inappropriate probiotic use may result in negative health consequences, including systemic or localized infections, metabolic disturbances, excessive immune stimulation in susceptible individuals, or transmission of antibiotic resistance genes [74, 75]. Clearly, there is a strong need for further research into the efficacy of probiotics currently being used in captive wildlife and development of host-specific probiotics.

Moving forward, there are some insights gained from this survey. First, the use of probiotics is quite widespread in wildlife facilities despite there being little evidence of their efficacy and the lack of species-specific products. Second, aside from antibiotics from clinically relevant antibiotic classes, antifungals and QACs are also commonly used. All of these antimicrobials need to be considered when assessing the emergence of AMR in wildlife settings and its potential spread into the environment. Finally, survey respondents stated that they would benefit from having standardized antimicrobial use guidelines in place. Quantifying the use of antimicrobials and probiotics, which is something this survey did not address, could be a relevant next step towards that goal. This should also include plans to monitor changes in antimicrobial and probiotic use over time to evaluate the effectiveness of any new implemented guidelines [44]. Additionally, more targeted training for veterinarians and wildlife professionals on AMU and probiotic use in wildlife rehabilitation would be beneficial. This would be especially helpful given the resource-limitations of many wildlife facilities and the varying degrees of direct veterinary involvement in the day-to-day medical care of wildlife patients. Ultimately, implementing use guidelines could improve therapeutic strategies for wildlife species while also promoting the prudent use of antibiotics and other microbial interventions.

## Supporting information

S1 File Complete survey. (PDF)
