# Supplementary material for: Survey of antimicrobial and probiotic use practices in wildlife rehabilitation in the United States
Source: PLoS One. 2024 Aug 1;19(8):e0308261. doi: 10.1371/journal.pone.0308261 (PMC11293748; doi:10.1371/journal.pone.0308261)
Supplement: S1 File — (PDF) [file pone.0308261.s001.pdf]

## Section 1: General Information (Questions 1-4)

**Antimicrobial and Probiotic Usage in Wildlife Rehabilitation**

Thank you so much for choosing to participate in our study. Your answers will remain **anonymous**.

The goal of our study is to **understand the current antimicrobial and probiotic use at rehabilitation centers** in the United States. The results of this survey will be used to understand the frequency and usage of antimicrobials and probiotics in wildlife rehabilitation. Your answers will help us in developing an antimicrobial stewardship program for wildlife rehabilitation.

“Antimicrobial” is an overarching term that includes any agent that kills microorganisms such as fungi, bacteria, viruses, or protozoa. “Probiotics” are live microorganisms, including bacteria and yeasts, that can confer a health benefit on the host when administered in appropriate amounts.

If any of the following protocols or lists are available, they will be helpful to have when you work on the survey:

- **Written protocols for antibiotic or antifungal use by a veterinarian.** Please refer to your facility's **most commonly used protocol**, we understand that individual antimicrobial use may be subject to clinical discretion by a veterinarian and will differ case by case.
- **List of antibiotics or antifungals** used at your facility
- **List of probiotics** used at your facility

- **List of disinfectants** used at your facility

1. What type of wildlife is treated at your facility? Check all that apply.

☐ Mammals
☐ Birds
☐ Reptiles
☐ Amphibians
☐ Fish
☐ Invertebrates
☐ ______ Other:

2. What type of birds do you treat at your facility? Check all that apply.

☐ Raptors (eagles, hawks, falcons, owls, ospreys, vultures)
☐ Small birds (songbirds, pigeons, turkeys)
☐ Waterfowl (ducks, geese, swans)
☐ Shorebirds (gulls, terns, pelicans)
☐ ______ Other:

3. How many bird admissions do you have in an average (pre-pandemic) year?

○ 0-10
○ 11-50
○ 51-100
○ 101-500
○ 501-1000
○ 1001-5000
○ 5001-10,000
○ Greater than 10,001

- ○ ______ Other:

4. Does your facility have access to one or more veterinarians?

- ○ Yes
- ○ No

**Section 2: Antibiotics and Antifungals (Questions 5-18)**

5. Do you use antimicrobials for treatment in your facility (This includes any of the following: antibiotic, antifungal, and antiviral medications)?

- ○ Yes
- ○ No

6. Do you have current written and documented protocols in place for antibiotic use made by a veterinarian?

- ○ Yes, for all circumstances
- ○ For some circumstances, not all
- ○ No
- ○ ______ Other:

7. Do you have current written and documented protocols in place for antifungal use made by a veterinarian?

- ○ Yes, for all circumstances
- ○ For some circumstances, not all
- ○ No

- ○ ☐ Other:

8. What percentage of your expenses contribute to antibiotics and antifungals?

- ○ 0-5%
- ○ 6-10%
- ○ 11-30%
- ○ 31-50%
- ○ 51-75%
- ○ Greater than 75%
- ○ Unknown

9. Are there any circumstances where bacterial culture and sensitivities are performed at your facility (either in-house or sent out to a diagnostic lab)?

- ○ Yes
- ○ No

10. Do you send these samples to a lab or perform them in-house?

- ○ Send out to lab
- ○ Perform in-house
- ○ Both, depends on the circumstance
- ○ ☐ Other:

11. What factors affect your facility's decision making concerning antibiotic or antifungal use? Check all that apply.

- ☐ Cost of antibiotic/antifungal
- ☐ Pre-made protocol by veterinarian

- ☐ Antimicrobial resistance
- ☐ Experience with drug
- ☐ Treatment success/ Response to treatment
- ☐ Duration of treatment
- ☐ Condition being treated
- ☐ Bacterial culture and sensitivity results
- ☐ ________ Other:

12. How often does your facility routinely use the following antibiotics? Listed as (Generic Name/Name Brand where applicable). If your facility uses one or more antibiotics that are not listed, please enter them in the "Other" box below.

| | Never | Rarely | Occasionally | Frequently | Very Frequently |
|---|---|---|---|---|---|
| Enrofloxacin/Baytril | ○ | ○ | ○ | ○ | ○ |
| Amoxicillin-clavulanic acid/Clavamox | ○ | ○ | ○ | ○ | ○ |
| TMPS (Trimethoprim / Sulfamethoxazole)/SMZ-TMP | ○ | ○ | ○ | ○ | ○ |
| Metronidazole/Flagyl | ○ | ○ | ○ | ○ | ○ |
| Gentamicin | ○ | ○ | ○ | ○ | ○ |
| Other: ________ | ○ | ○ | ○ | ○ | ○ |

13. For each of the following circumstances, please select the percentage of cases your facility treats with antibiotics (assume uncomplicated and primary presentation). Please refer to your facility's **most commonly used protocol.**

| | 0% Our facility does not use antibiotics for this circumstance | 25% or fewer | 26-50% | 51-75% | 76-99% | 100% Our facility uses antibiotics for all instances of this circumstance |
|---|---|---|---|---|---|---|
| Bite Wounds | | | | | | |
| Orphans | | | | | | |
| Window strike | | | | | | |
| Open fractures | | | | | | |
| Emaciation | | | | | | |
| Projectile (gun shot) | | | | | | |
| Electrocution or burns | | | | | | |
| Contaminant (i.e. oil, glue trap) | | | | | | |
| Suspect toxicity (i.e. lead, rodenticide) | | | | | | |
| Prophylaxis or prevention of secondary infection | | | | | | |
| Other: | | | | | | |

14. How often does your facility routinely use the following antifungals? Listed as (Generic Name/Name Brand where applicable). If your facility uses one or more antifungals that are not listed, please enter them in the "Other" box below.

| | Never | Rarely | Occasionally | Frequently | Very Frequently |
|---|---|---|---|---|---|
| Nystatin/Mycostatin | | | | | |
| Itraconazole/Itrafungol | | | | | |
| Voriconazole/Vfend | | | | | |
| Terbinafine/Lamisil | | | | | |
| Other: | | | | | |

15. For each of the following circumstances, please select the percentage of cases your facility treats with antifungals (assume uncomplicated and primary presentation). Please refer to your facility's **most commonly used protocol.**

| | 0% Our facility does not use antifungals for this circumstance | 25% or fewer | 26-50% | 51-75% | 76-99% | 100% Our facility uses antifungals for all instances of this circumstance |
|---|---|---|---|---|---|---|
| Prophylaxis or prevention of secondary infection | ○ | ○ | ○ | ○ | ○ | ○ |
| Emaciation | ○ | ○ | ○ | ○ | ○ | ○ |
| Respiratory disease | ○ | ○ | ○ | ○ | ○ | ○ |
| Wounds or lacerations | ○ | ○ | ○ | ○ | ○ | ○ |
| Other: ______ | ○ | ○ | ○ | ○ | ○ | ○ |

16. What factors affect your facility’s decision making concerning stopping antibiotic or antifungal use? Check all that apply.

- ☐ Cost of antibiotic/antifungal
- ☐ Pre-made protocol by veterinarian with set time frame
- ☐ Antimicrobial resistance
- ☐ Adverse reaction to drug
- ☐ Resolution of clinical signs
- ☐ Recommended duration of drug (i.e. 10-14 days)
- ☐ Culture and sensitivity results
- ☐ ______ Other:

17. Where does your facility get your information about antibiotics or antifungals and their usage? Check all that apply.

- ☐ Veterinarians
- ☐ Other rehabilitators
- ☐ Continuing education courses
- ☐ Peer reviewed literature (i.e. scientific journal, textbook, reference manual, etc.)
- ☐ Internet
- ☐ Social media
- ☐ ________ Other:

18. Tell us how strongly you agree or disagree with the following statements on antimicrobial resistance (AMR):

| | Strongly agree | Somewhat agree | Neither agree nor disagree | Somewhat disagree | Strongly disagree |
|---|---|---|---|---|---|
| Our facility would benefit from standardized antimicrobial and antifungal use guidelines. | ○ | ○ | ○ | ○ | ○ |
| Our facility has a general knowledge of AMR in relation to the antibiotics and antifungals we use. | ○ | ○ | ○ | ○ | ○ |
| AMR is (or has been) a problem at our facility. | ○ | ○ | ○ | ○ | ○ |
| Our facility believes there is a high risk for AMR in the field of wildlife rehabilitation. | ○ | ○ | ○ | ○ | ○ |

**Section 3: Disinfectants (Questions 19-22)**

19. Does your facility use any disinfectants to clean the facility?

- ○ Yes
- ○ No

20. Do you use any of these Quaternary Ammonium compounds? Check all that apply.

- ☐ Roccal D™
- ☐ Parvosol™
- ☐ Quinticide™
- ☐ ____________ Other
- ☐ We do not use any of these options

21. For what purpose does your facility use these disinfectants (from Question 20)?

- ○ Cleaning of surfaces
- ○ Cleaning of kennels/cages/animal containment areas
- ○ Cleaning of wounds
- ○ ____________ Other:

22. How often does your facility use these disinfectants?

- ○ Daily
- ○ Weekly
- ○ Monthly
- ○ ____________ Other:

## Section 4: Probiotics (Questions 23-30)

23. Do you use any probiotics in your facility?

- ○ Yes
- ○ No

24. How often does your facility routinely use the following probiotics? If your facility uses one or more probiotics that are not listed, please enter them in the "Other" box below.

| | Never | Rarely | Occasionally | Frequently | Very Frequently |
|---|---|---|---|---|---|
| Purina® Pro Plan® Fortiflora | ○ | ○ | ○ | ○ | ○ |
| Plain yogurt (any brand) | ○ | ○ | ○ | ○ | ○ |
| Pet Ag® Bene-bac Plus | ○ | ○ | ○ | ○ | ○ |
| Neckton-Biotic-Bird | ○ | ○ | ○ | ○ | ○ |
| Fox Valley Probiotics | ○ | ○ | ○ | ○ | ○ |
| Other: ____ | ○ | ○ | ○ | ○ | ○ |

25. For each of the following circumstances, please select the percentage of cases your facility treats with probiotics (assume uncomplicated and primary presentation). Please refer to your facility's **most commonly used protocol.**

| | 0% Our facility does not use probiotics for this circumstance | 25% or fewer | 26-50% | 51-75% | 76-99% | 100% Our facility uses probiotics for all instances of this circumstance |
|---|---|---|---|---|---|---|
| On all animals | ○ | ○ | ○ | ○ | ○ | ○ |

| | 0% Our facility does not use probiotics for this circumstance | 25% or fewer | 26-50% | 51-75% | 76-99% | 100% Our facility uses probiotics for all instances of this circumstance |
|---|---|---|---|---|---|---|
| Emaciation | ○ | ○ | ○ | ○ | ○ | ○ |
| Diarrhea | ○ | ○ | ○ | ○ | ○ | ○ |
| Constipation/absence of feces or mutes | ○ | ○ | ○ | ○ | ○ | ○ |
| Alongside with antibiotic use | ○ | ○ | ○ | ○ | ○ | ○ |
| As a calming agent | ○ | ○ | ○ | ○ | ○ | ○ |
| Other: ______ | ○ | ○ | ○ | ○ | ○ | ○ |

26. Tell us how strongly you agree or disagree with the following statement:

| | Strongly agree | Somewhat agree | Neither agree nor disagree | Somewhat disagree | Strongly disagree |
|---|---|---|---|---|---|
| Probiotics improve a patient’s clinical outcome. | ○ | ○ | ○ | ○ | ○ |
